# Evaluation of the Effects of Skin-to-Skin Contact on Newborn Sucking, and Breastfeeding Abilities: A Quasi-Experimental Study Design

**DOI:** 10.3390/nu14091846

**Published:** 2022-04-28

**Authors:** Jia-Zhen Huang, Chi-Nien Chen, Chih-Ping Lee, Chien-Huei Kao, Heng-Cheng Hsu, An-Kuo Chou

**Affiliations:** 1 Department of Nursing, National Taiwan University Hospital Hsin-Chu Branch, Hsin-Chu 30059, Taiwan; kk7771kimo@gmail.com (J.-Z.H.); chplee@hch.gov.tw (C.-P.L.); 2 Department of Nurse-Midwifery and Women Health, National Taipei University of Nursing and Health Sciences, Taipei 112303, Taiwan; 3 Department of Pediatrics, National Taiwan University Hospital Hsin-Chu Branch, Hsin-Chu 30059, Taiwan; dtped124@gmail.com; 4 Department of Obstetrics and Gynecology, National Taiwan University Hospital Hsin-Chu Branch, Hsin-Chu 30059, Taiwan; b101092037@gmail.com

**Keywords:** breastfeeding, skin-to-skin contact, feeding ability, breastfeeding self-efficacy, GEE modeling

## Abstract

Mother and newborn skin-to-skin contact (SSC) after birth has numerous protective effects. Although positive associations between SSC and breastfeeding behavior have been reported, the evidence for such associations between early SSC and breastfeeding success was limited in high-income countries. This quasi-experimental intervention design study aimed to evaluate the impact of different SSC regimens on newborn breastfeeding outcomes in Taiwan. In total, 104 healthy mother–infant dyads (52 in the intervention group and 52 in the control group) with normal vaginal delivery were enrolled from 1 January to 30 July 2019. The intervention group received 60 min of immediate SSC, whereas the control group received routine care (early SSC with 20 min duration). Breastfeeding performance was evaluated by the IBFAT and BSES-Short Form. Generalized estimating equations (GEEs) were used to evaluate the effectiveness of the intervention. In the intervention group, the breastfeeding ability of newborns increased significantly after 5 min of SSC and after SSC. The intervention also improved the total score for breastfeeding self-efficacy (0.18 point; *p* = 0.003). GEE analysis revealed that the interaction between group and time was significant (0.65 point; *p* = 0.003). An initial immediate SSC regimen of 60 min can significantly improve neonatal breastfeeding ability and maternal breastfeeding self-efficacy in the short term after birth.

## 1. Introduction

Breastfeeding has long-term health benefits for mothers and children, and knowledge of ways to improve breastfeeding can increase the chances of breastfeeding success [1]. Early mother–infant skin-to-skin contact (SSC) is a key factor in the success of breastfeeding [1,2,3]. SSC also has other benefits, such as reduced mortality among low-birth-weight infants [4].

A national survey in Taiwan found that the prevalence of SSC reached more than 60%, and early SSC had a significant impact on exclusive breastfeeding success up to 6 months [5]. In addition, SSC can help reduce early hospitalization rates after birth [6]. Previous studies have found that breastfeeding success is related to the duration of SSC, and longer duration may positively correlate with greater breastfeeding rates [7]. The World Health Organization recommends that SSC between mother and baby be performed for more than an hour after birth [8]. In contrast, the mother- and infant-friendly medical institution regulation in Taiwan states only that early SSC is encouraged and that an SSC duration of at least 20 min is recommended. The definitions for the duration of SSC and the exact timing for the initiation of SSC differed in previous studies and were also heterogeneous [9].

There are limited studies on the SSC duration needed for it to be effective, and most of these studies were conducted in low-income countries [2,3,7,10]. Although skin-to-skin contact was positively associated with breastfeeding behavior in many countries, a cross-sectional study found no association between early SSC and breastfeeding success in Australia [11]. The breastfeeding outcomes after SSC in high-income countries are controversial, and the prevalence rates of SSC in high-income countries were mostly over 50~60% in a systematic review [9]. It is worth exploring whether the duration of SSC can enhance breastfeeding success in high-income countries and whether there is a dose–response relationship.

Many concerns have not been addressed yet, probably due to the lack of a consistent definition for SSC initiating time or duration in the current clinical practice setting. There are insufficient randomized studies related to SSC and breastfeeding outcomes to support the evidence [3,10,12]. A study in Colombia found that SSC immediately after birth was not associated with better breastfeeding outcomes than early skin-to-skin contact at 60 min after birth [13]. Whether there are no differences in the onset time of SSC in high-income countries remains to be confirmed.

Given the many advantages of SSC, there are ethical concerns about using randomized controlled studies to understand the effect of SSC. Thus, the comparison of the immediate and prolonged SSC with routine neonatal care in Taiwan might be an implementable project for exploration. We hypothesized that immediate onset of SSC and longer duration may promote the later breastfeeding ability in neonates. To test this hypothesis, this study aimed to investigate whether the breastfeeding outcomes are affected by SSC for different onset times and durations through a quasi-experimental study design.

## 2. Materials and Methods

### 2.1. Study Participants

We conducted a single-centre study using a quasi-experimental intervention design to evaluate whether immediate and longer SSC had an impact on newborn sucking, feeding, and breastfeeding efficiency. We have followed the TREND checklist to report our study [14].

Women who had undergone natural childbirth in the delivery room in the National Taiwan University Hospital Hsin-Chu Branch, a regional teaching hospital with approximately 700 births annually and their newborns were recruited as the study participants. Sample size estimation was performed with a moderate effect size of 0.25 and α error of 0.05 and achieved 80% power for the study. Consequently, the sample size was estimated to be 52 mother–infant dyads and a total of 104 pairs in both the intervention and control groups.

From 1 January to 30 July 2019, the following two phases were performed with 2 weeks suspended between these two phases: (1) the first phase with the participation of mother–infant dyads born from 1 January to 28 March 2019, serving as the control group, and (2) the second phase including a training program for neonatal nurses to optimize their clinical practices and with the participation of mother–infant dyads born from 11 April to 30 July 2019, serving as the intervention group. The mother–newborn pairs in the control group had continuous skin contact for 20 min according to original routine neonatal care (for about five to ten minutes) at the institution. Regarding neonatal care before SSC in the control group, we conducted umbilical cord cutting, birth weight, head circumference, birth height, and body temperature measurements. The umbilical cord-cutting was conducted at the same time in both groups. Neonates who received routine nursing care after birth had a significantly longer time to initiate breastfeeding than those who received uninterrupted and immediate SSC [15]. In contrast, those in the intervention group had uninterrupted skin contact for 60 min immediately after birth.

The criteria for inclusion in this study were vaginal delivery, singleton pregnancy at 37–42 gestational weeks, infant birth weight between 2500 and 4000 g, and the ability of the mother to listen, speak, read, and write in Chinese. Women awaiting delivery gave informed consent to participate in the study and agreed to have postpartum mother–infant skin contact. The exclusion criteria were medical and obstetric complications due to a high-risk pregnancy; refusal to breastfeed or choosing to use formula for feeding prior to delivery; interrupted skin contact between the mother and infant for medical reasons, such as postpartum hemorrhage or perineal lacerations; breast structural abnormalities that could affect neonatal sucking, such as flat or inverted nipples; a need for the newborn to be admitted to the infant observation room or neonatal intensive care ward immediately after birth; first Apgar score less than 7 and congenital malformations, such as cleft lip. Nipple problems may affect breastfeeding success [16]; therefore, we excluded women with flat or inverted nipples.

### 2.2. Research Tools and Outcome Definitions

The primary outcomes were newborn sucking, breastfeeding ability, and breastfeeding self-efficacy. The research tools used consisted of (1) a questionnaire for collecting basic information about the mother and neonate, (2) the infant breastfeeding assessment tool (IBFAT) [17], and (3) the Breastfeeding Self-Efficacy Scale-Short Form (BSES-SF) [18]. The research tools utilized in the study were all authorized by the original authors. Both of these two tools have been used by many scholars in Taiwan before. In addition, there are also Chinese versions of these two tools for study with good reliability, which can assist in the conduction of this study [19,20]. To evaluate breastfeeding ability, four consecutive IBFAT scores were collected at the following times: (1) after 5 min of SSC, (2) after skin contact (about 25 to 30 min of age in the control group and 60 min of age in the intervention group), (3) 24 h after birth, and (4) before discharge (3 days of age). Maternal breastfeeding self-efficacy was assessed by the BSES-SF on the day of discharge. We used the above questionnaires to analyse the performance after SSC. The secondary outcome was the success rate of breastfeeding after SSC.

### 2.3. Training of Nursing Staff

A total of 16 nursing staff members were recruited as data collectors. The data collectors were all personnel who had at least 3 years of nursing experience in the obstetrics-postpartum ward. Consensus meetings were held before data collection to ensure consistency between data collectors when using tools for infant breastfeeding evaluation. Consensus meetings also included training of the staff, which included the following: (1) Guiding skin contact health education, (2) Codifying the conditions and execution times of the intervention, and (3) Watching video recordings of infant breastfeeding for joint evaluation. Scoring was based on the IBFAT. The presence of breast structural abnormalities, such as flat or inverted nipples, was discovered to potentially affect neonatal sucking ability, according to pictures captured from three different visual angles of the breast. Consistency of data collection reached 96% before the actual study was initiated. The study purpose, content, and requirements were discussed with the hospital. Patients who fit the inclusion criteria were recruited after they had provided consent. Data collection did not commence until the consent form was signed by the patient or her spouse. This study was reviewed and approved by the Ethics Committee for Human Trials of National Taiwan University Hospital Hsin-Chu Branch (No. 107-061-E) and registered at ClinicalTrials.gov (NCT04142099).

### 2.4. Statistical Analysis

Continuous variables were summarized and presented as the means (standard deviation, SD); categorical variables were summarized and are presented as frequencies and percentages. The collected data, including number distributions, percentages, average values, Student’s *t*-test and chi-squared test, were analyzed using descriptive and inferential statistics according to the research purpose and characteristics.

Generalized estimating equation (GEE) modeling was used to determine factors related to the primary outcome, such as the sucking ability and breastfeeding self-efficacy scales [21]. GEE modeling is widely used for the analysis of longitudinal data with repeated measurements on the same subjects over time. We utilized GEE modeling to evaluate the effect between the intervention and control groups with different time points of IBFAT measurements. Newborn sucking ability and breastfeeding self-efficacy between the intervention and control groups were also assessed. A *p*-value less than 0.05 was considered statistically significant, and the statistical analyses were performed using SPSS version 23.0 (IBM Corp., Armonk, NY, USA)

## 3. Results

### 3.1. Clinical Characteristics of Study Participants

**Figure 1:**
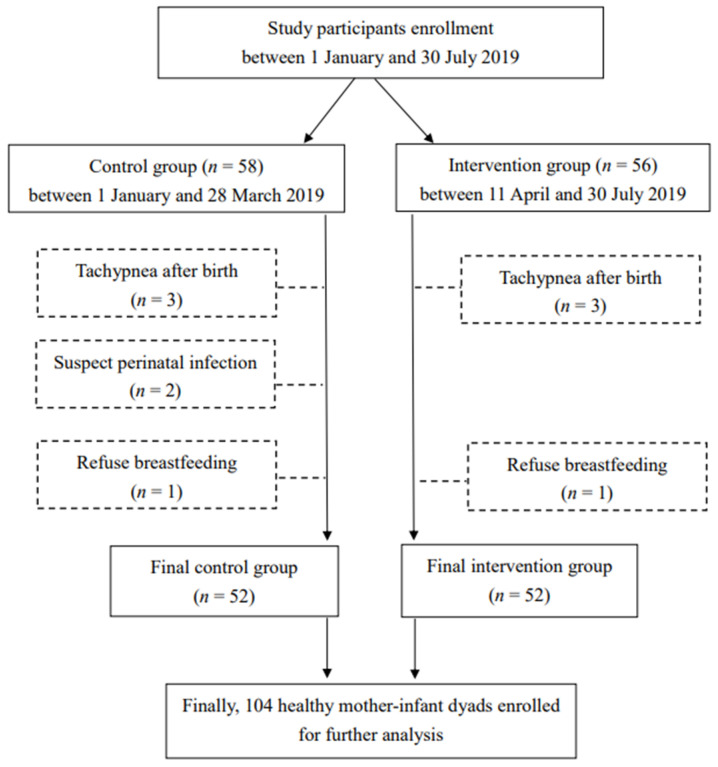
The flow of study participants.

The flow of study participants is illustrated in Figure 1. This study included 104 mother–infant dyads (52 in the intervention group and 52 in the control group) from 1 January to 30 July 2019. The clinical characteristics of the study participants are summarized in Table 1 and Table 2. The baseline clinical parameters were comparable between the intervention and control groups. There were no significant differences in the maternal characteristics, including the maternal age at childbearing, maternal educational status, marital status, employment status, parental leave, previous infant feeding experiences, awareness of SSC, pain control, parity, and delivery assistance methods (Table 1). Regarding the neonatal characteristics, there were also no significant differences in the gestational age at delivery, infant sex, anthropometric measurements at birth, or Apgar scores at 1 and 5 min after birth (Table 2).

### 3.2. Analysis of the Effect between the Intervention and Control Groups on Newborn Breastfeeding Ability

Four consecutive IBFAT scores, namely, after 5 min of SSC, after skin contact, 24 h after birth, and before discharge, were collected (Table 3). The results indicated that 15 (28.8%) neonates in the control group successfully breastfed. In the comparisons of breastfeeding ability between the newborns in the control and intervention groups at different time points, our study findings revealed that their IBFAT scores were, respectively, 1.19 ± 0.99 and 1.85 ± 1.27 (*p* < 0.05) after 5 min of SSC and 3.23 ± 2.38 and 6.37 ± 2.47 (*p* < 0.05) after skin contact had ended.

**Figure 2:**
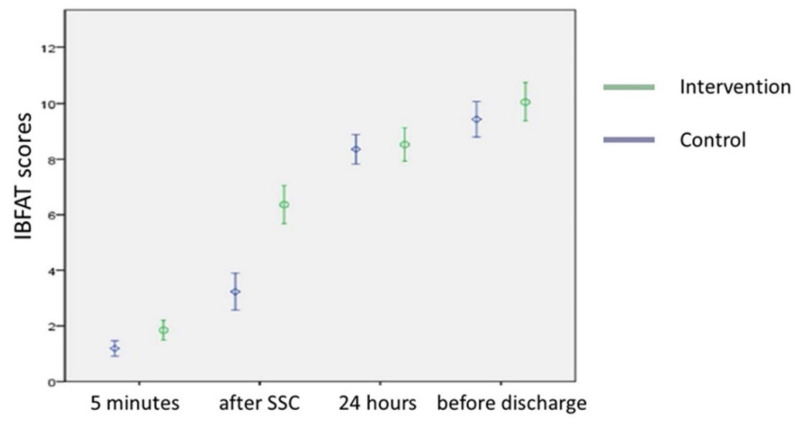
Evaluation of newborn breastfeeding ability at different time points between the intervention and control groups (data are presented as the median with 95% CI).

In terms of newborn breastfeeding ability after 5 min of SSC, until skin contact had ceased, neonates in the intervention group had a moderately effective sucking ability. At 24 h after birth and before discharge, there was no significant difference between the two groups. Evaluation at different time points indicated that the breastfeeding ability and sucking ability scores for both groups of newborns increased with time (Figure 2). These results show that a skin contact time of 60 min between the mother and infant in the intervention group resulted in a higher average score.

Table 4 summarizes the results from the longitudinal analysis evaluating the main and interaction effects of the intervention on the IBFAT scores over time by GEE modeling. Regarding the main effect (group effect), the GEE model revealed that the mean scores for the sucking ability of newborns differed significantly between the control group and intervention group after 5 min of SSC, after skin contact, at 24 h after birth, and before discharge. The main effect analysis revealed significantly increased neonatal sucking ability in the intervention group compared with the control group (1.18 points; *p* < 0.001).

Regarding the interaction effect (group*time), total scores on the breastfeeding self-efficacy scales were tested as covariates, and the interaction between groups and times was evaluated. The results revealed that the average scores for the sucking ability of newborns in the two groups were significantly higher (2.48 points; *p* < 0.001) after skin contact than after 5 min of SSC. For all newborns, the average scores for neonatal sucking ability increased after skin contact by 2.04, 7.17, and 8.25 points after skin contact, 24 h after birth, and before discharge, respectively. The effect of intervention on IBFAT differed over time (0.65 points; *p* = 0.003). Our results demonstrate that in the intervention group, neonatal sucking ability increased with the duration of skin contact and that after SSC, it was significantly different from the score after 5 min of SSC (*p* < 0.001); however, the average score did not increase after the end of skin contact in the control group.

### 3.3. Analysis of the Effect between the Intervention and Control Groups on the Self-Efficacy of Breastfeeding

After controlling the “total breastfeeding self-efficacy score” variable as a covariate, we verified the detection effect analysis by GEE modeling. The findings indicated that the intervention group had 0.18 point higher self-efficacy scores than the control group, which may correspond to improved neonatal sucking scores (*p* = 0.003; Table 4).

## 4. Discussion

### 4.1. Main Findings

To our knowledge, this is the first published prospective study to examine the effect of SSC intervention for 1 h immediately after birth on breastfeeding ability and self-efficacy outcomes compared with routine care using a quasi-experimental design in Taiwan. Our study findings indicated that most newborns who were exposed to the intervention group (*n* = 35, 67.3%) had successful breastfeeding after SSC, whereas only 15 (28.8%) did in the control group. Notably, we used the GEE approach to reveal that the interaction between group and time was significant (0.65 points; *p* = 0.003), which means that the effect of the intervention on IBFAT differed over time. In addition, mothers in the intervention group experienced better breastfeeding self-efficacy. In line with previous literature, our study confirms that SSC for 1 h after birth may lead to better breastfeeding performance. One multi-country cross-sectional observational study in Asia and the Pacific found a dose–response impact on breastfeeding outcomes by longer SSC. The odds ratio for exclusive breastfeeding by SSC for 60–89 min after birth was 5.61 compared with women without SSC [7]. Concurrent implementation of immediate uninterrupted and longer duration of SSC after birth enables us to see the impact of better sucking ability in the intervention group.

Although SSC was positively associated with breastfeeding behavior in many countries, a cross-sectional study found no association between early SSC and breastfeeding behavior in Australia [11]. Whether the effects of SSC are the same in high-income countries warrants further study. Our study also found that the effect of early SSC on breastfeeding was significant mainly at the initial stage, but there was no significant difference before discharge from the hospital.

### 4.2. Effect of the SSC Intervention on Newborn Breastfeeding Ability

Whether the long-term benefits were sustainable or limited in the early stage needs to be considered in many aspects. Our results differ from those of a clinical randomized trial study conducted by Mahmood, Jamal, and Khan in Pakistan on SSC and breastfeeding [10]. In our study, the rate of successful lactation was higher in the intervention group than in the control group. A crucial insight is that newborn breastfeeding ability is related to an innate instinct [22]. Although neonates in the control group were also able to breastfeed effectively and successfully after a brief period of skin contact, the present study found that the number of effectively breastfeeding neonates was higher after a prolonged period of 60 min of mother–infant skin contact.

Whether immediate and uninterrupted SSC could provide better newborn breastfeeding ability needs to be further clarified. In one randomized clinical trial in Colombia, no differences in the IBFAT scores were observed between the immediate and early SSC groups [13]. The authors proposed that the nondifference in the neonatal breastfeeding indicators could be explained by the existing well breastfeeding education and training established in their institution. In addition, they supposed that the onset times of SSC during the neonatal sensitive period could achieve acceptable oxytocinergic system activation for breastfeeding success. However, they defined “early” as SSC at 60 min of life, which differed from our study (5 to 10 min after basic neonatal care). This may be the main reason for the inconsistent results.

According to the IBFAT [17], the breastfeeding ability of newborns gradually develops during the days after birth. Newborns must learn to breastfeed in the first few days to help meet their physiological needs. Delays in breastfeeding can occur among newborns who are required to undergo further evaluation for pathological aetiologies after birth, taking an average of 30–36 h, with extremes of up to 40–80 h. The length of time required is related to individual physiological differences and the mother’s breastfeeding skills. This accords with our finding that newborns in the intervention group who experienced 60 min of mother–infant skin contact, with a longer time to familiarize themselves with the breasts, displayed favorable breast-seeking and breastfeeding behavior.

### 4.3. Effect of the SSC Intervention on Postpartum Feeding

In our study, the rate of exclusive breastfeeding in both groups decreased with the number of days after birth (Table 3). Chiou et al. [23] found that breastfeeding experience and early SSC after delivery tend to promote postpartum breastfeeding and increase the rate of exclusive breastfeeding during hospitalization. This finding differs from the results of the present study. Possible reasons include that the study participants came from different sources. We enrolled hospitalized mothers into the intervention and control groups for analysis. Their study used a nationally representative sample for analysis in 2004 and 2011. At that time, the promotion of breastfeeding and the willingness to breastfeed may not be the same as in our study, which may cause differences in the study results. Of all the participants, most of them had ever heard of SSC or had interventions, such as pain-relieving methods, use of facilitating drugs, and use of assistive devices applied during labor. Postpartum fatigue is also associated with breastfeeding success [24]. Such approaches to medical delivery may contribute to postpartum fatigue. In addition, postpartum women need time to recover and adapt to their new roles [25]; thus, mothers in this study may have changed their feeding style.

Bramson et al. [26] reported that 1–3 h of mother–infant skin contact could effectively improve breastfeeding and lead to exclusive breastfeeding during a hospital stay. This result conflicts with our findings, possibly because of differences in the study sample size. In addition, the underlying social support factor is also an important influence on breastfeeding success and deserves further exploration. Family support systems are correlated with mothers’ methods of breastfeeding after delivery when confronted with neonatal feeding problems [27]. Insufficient family support affects the mother’s feeding styles, leading to mixed feeding approaches during hospitalization or even cessation of breastfeeding [28]. In the present study, an analysis of postpartum breastfeeding styles and influential factors pertaining to family members indicated that 22 (42.3%) participants in the intervention group and 19 (36.5%) in the control group supported breastfeeding, with no significant difference noted between the two groups. This finding also reflects the current public health policy to encourage breastfeeding, which is also supported by family members in the current trend. This may also result in limited changes or impacts after a longer SSC duration under a relatively high SSC prevalence rate in Taiwan.

Family support plays an important role in the success of breastfeeding in Taiwan [29]. Lack of support for exclusive breastfeeding may be due to the small amount of milk secreted during the early postpartum period, fear of insufficient breast milk and the inability to meet the needs of the baby, exhaustion and lack of physical breastfeeding after delivery, the feeding approach, and rapid weight loss of the newborn, which is expected during physical recovery. Some family members may think that breast milk is not enough and will make the baby hungry or make the newborn present with persistent hyperbilirubinemia. They will not support the mothers to continue breastfeeding. During this time, family members may attempt to persuade mothers to use mixed feeding approaches for the newborn. While learning how to breastfeed, any resistance or difficulties evoke negative emotions in the mother. Negative emotions may arise from concerns that the mother’s milk supply is insufficient, thus interfering with oxytocin secretion, which affects milk secretion. Lack of family support for breastfeeding may cause mothers to change their attitudes and choose not to adopt exclusive breastfeeding [30]. In addition to promoting skin-to-skin contact to help the effectiveness of breastfeeding, clinical practitioners also have to enhance the educational program to increase family members’ support for breastfeeding.

### 4.4. Effect of the SSC Intervention on Breastfeeding Self-Efficacy

Our results indicate that breastfeeding self-efficacy has a significant positive correlation with the breastfeeding ability of newborns. The BSES-SF showed good reliability and validity to assess mothers’ confidence to breastfeed infants among Mandarin-Speaking Chinese Mothers [31]. In other words, higher levels of confidence in breastfeeding among mothers lead to higher breastfeeding ability among newborns. This study employed an intervention with a prolonged mother–infant skin contact time of 60 min compared with routine care and revealed that mothers in the intervention group had higher breastfeeding self-efficacy scores. However, mothers completed the self-efficacy questionnaire on the day of discharge, meaning that the results may have been skewed by the encouragement and care that postpartum women received from medical staff during hospitalization or visits and messages received from relatives and friends. We suggest that this should be considered in future studies. The self-administered breastfeeding self-efficacy questionnaire can be administered to the study participants after skin contact and again on the day of discharge to examine the results at different time points. We found a statistically significant positive correlation between breastfeeding self-efficacy and neonatal lactation. This accords with the findings of Awaliyah et al. [32] that mothers who breastfed as early as possible after delivery had higher breastfeeding self-efficacy scores. Thus, the uninterrupted and immediate 60-min skin contact intervention likely not only improved the breastfeeding ability of newborns but also enhanced the breastfeeding self-efficacy of mothers, suggesting that adopting this approach for routine clinical care in Taiwan is worthwhile.

### 4.5. Potential Explanatory Mechanisms for the Benefits from SSC

There are many possible factors involved in the mechanism underlying the effect of SSC on breastfeeding success that deserve further exploration. The most well-known of these are attributed to the physiological feedback mechanism of the oxytocinergic system [33]. SSC of newborns crawling on their mothers could stimulate oxytocin release in mothers [34]. The rise of oxytocin in mothers could also inhibit the sympathetic nervous system response, causing peripheral blood vessels to dilate, increasing the body surface temperature and helping the newborn to control body temperature [35]. This mechanism has also been confirmed in animal experiments [36]. These are important mechanisms that help newborns survive in the transitional stage after birth. Therefore, a longer duration of SSC may provide better health outcomes in neonates with a dose–response effect.

### 4.6. Strengths and Limitations

The strength of this study is its use of a prospective quasi-experimental design to observe that the intervention group with 60 min of mother–infant SSC had better breastfeeding outcomes than the control group. The causal relationship needs to be verified. The utilization of the GEE statistical method can effectively carry out the comparison of repeated measures, providing more robust evidence.

Although this study has provided rich information to explain the effect of SSC with a dose–response impact by a prospective quasi-experimental study design, our study findings should be viewed in light of the following limitations. First, possible socioeconomic effects should be considered in the study, but the number of cases in this study is not sufficient for subsequent regression analysis or subgroup study to understand the effects of other confounding factors. Second, we collected only the outcome data before discharge, and the long-term impact of SSC should be evaluated. Third, what precise onset time or SSC duration is best? Further research on whether immediate or early and 90 or 120 min of SSC may be better for the prognosis of offspring and if there is a risk of adverse effects is warranted. Fourth, the measurement time points after SSC differed, and the IBFAT scores might be affected by the age after birth. We cannot compare the two groups simultaneously after 5 min of SSC and after SSC because the control group had an additional five to ten minutes of neonatal care. The SSC duration differed in the control and intervention groups. In the control group, the measurement time point after SSC was close to half an hour after birth, but the time point in the intervention group was one hour old. The comparison of early SSC effects in future research design will need to consider this pitfall and design a feasible research method to solve this dilemma. Fifth, we used a quasi-experimental study design, and we cannot enroll cases simultaneously. So, the timing of study participants’ enrolment in the intervention group and the control group completed in the different months, might be a limitation affecting the research results. Finally, this study analyzed only low-risk vaginal-birth cases, so whether early SSC can be widely extended to high-risk cases, such as preterm birth, multiple births, cesarean delivery, or women with perinatal complications is unclear. Implementation needs to be clarified. Extending this intervention to high-risk groups will be an important prospect for future research.

## 5. Conclusions

The present study confirms that the initial SSC regimen of 60 min can significantly improve neonatal breastfeeding ability and maternal breastfeeding self-efficacy. Our study indicated the dose impact of SSC duration on breastfeeding ability. However, this benefit seems significant after 5 min of SSC and after SSC, and it diminished before discharge from the hospital. Evaluations of the long-term outcomes of SSC for 60 min are needed. Currently, we should prioritize the training of clinical nursing staff on the implementation of SSC and understand the benefits of SSC and the early initiation of breastfeeding. Future studies on SSC support in preterm infants or women who receive cesarean section require further investigation to demonstrate its benefits. Healthcare providers should provide early SSC for at least 60 min to improve breastfeeding success and promote various health benefits for mothers and their children.

## Figures and Tables

**Table 1:** Demographic characteristics of mothers.

Variables	Intervention (*n* = 52)	Control (*n* = 52)	*p* Value ^†^
Maternal age (years)	33.6 ± 5.3	32 ± 4.8	0.11
Parity			0.32
1	24 (46.2%)	23 (44.2%)	
2	17 (32.7%)	23 (44.2%)	
3 or more	11 (21.1%)	6 (11.6%)	
Maternal education			0.31
College or above	40 (76.9%)	45 (86.5%)	
Marital status			1
Married	51 (98.1%)	51 (98.1%)	
Employment status			1
Employed	38 (73.1%)	38 (73.1%)	
Parental leave			0.3
Yes	20 (38.5%)	15 (28.8%)	
Breastfeeding experience			0.85
Yes	28 (53.8%)	27 (51.9%)	
Ever heard of SSC			0.49
Yes	49 (94.2%)	46 (88.5%)	
Pain control			0.83
Yes	21 (40.4%)	20 (38.5%)	
Delivery assistance			0.21
Medication	39 (75%)	45 (86.5%)	

Data are presented as the number (%) or mean ± SD. ^†^ *p*-value was calculated by chi-square test, Fisher’s exact test, or *t*-test.

**Table 2:** Demographic characteristics of neonates.

Variables	Intervention (*n* = 52)	Control (*n* = 52)	*p* Value ^†^
Gestational age			0.23
37–38 weeks	25 (48%)	19 (36.5%)	
39–40 weeks	27 (52%)	33 (63.5%)	
Male sex	23 (44.2%)	28 (53.8%)	0.33
Birth weight (gm)	3102 ± 301	3114.2 ± 287.2	0.83
Birth body height (cm)	50.9 ± 1.7	51.1 ± 1.3	0.7
Apgar score (1 min)	9 (1)	9 (1)	0.38
Apgar score (5 min)	10 (1)	10 (1)	1

Data are presented as the number (%), median (IQR, interquartile range) or mean ± SD. ^†^ *p*-value was calculated by chi-square test, Fisher’s exact test, or *t*-test.

**Table 3:** Assessment of newborn sucking ability, breastfeeding self-efficacy, and exclusive breastfeeding rate.

Variables	Intervention (*n* = 52)	Control (*n* = 52)	*p* Value ^†^
IBFAT score			
After 5 min of SSC	1.85 ± 1.27	1.19 ± 0.99	0.005
After skin contact	6.37 ± 2.47	3.23 ± 2.38	<0.001
24 h after birth	8.54 ± 2.17	8.37 ± 1.92	0.48
Before discharge	10.06 ± 2.4	9.44 ± 2.28	0.15
BSES-SF score			
Before discharge	3.01 ± 0.84	3.1 ± 0.91	0.19
Successful breastfeeding after SSC			
Yes	35 (67.3%)	15 (28.8%)	<0.001
Exclusive breastfeeding rate			
First day of birth	33 (63.5%)	25 (48.1%)	0.12
Second day of birth	18 (34.6%)	17 (32.7%)	0.84
Before discharge	16 (30.8%)	15 (28.8%)	0.83

Data are presented as the number (%) or mean ± SD. ^†^ *p*-value was calculated by chi-square test, Fisher’s exact test, or *t*-test.

**Table 4:** Newborn sucking ability score comparison at different time points by the GEE approach.

Parameters	GEE1 (Main Effect)			GEE2 (Interaction)		
	ꞵ	SE	*p* Value	ꞵ	SE	*p* Value
Group effect (Intervention vs. control)	1.18	0.28	<0.001	0.65	0.22	0.003
After skin contact vs. after 5 min of SSC	3.28	0.22	<0.001	2.04	0.29	<0.001
24 h after birth vs. after 5 min of SSC	6.93	0.22	<0.001	7.17	0.3	<0.001
Before discharge vs. after 5 min of SSC	8.23	0.25	<0.001	8.25	0.35	<0.001
Group*time						
After skin contact vs. after 5 min of SSC				2.48	0.38	<0.001
24 h after birth vs. after 5 min of SSC				−0.48	0.45	0.29
Before discharge vs. after 5 min of SSC				0.08	0.48	0.87
Control variable						
Breastfeeding self-efficacy scale scores				0.18	0.17	0.003

Abbreviations: GEE, generalized estimating equation; GEE1: main effect of group and time; GEE2: interaction of group and time; SE: standard error.

## Data Availability

The data presented in this study are available on request from the corresponding author. The data are not publicly available due to patient confidentiality.
